# CONNECTING LIFETIME EXPERIENCE OF MISCARRIAGE TO POSITIVE LIFE EVALUATION AMONG WOMEN AGING-IN-CUSTODY

**DOI:** 10.1093/geroni/igad104.2166

**Published:** 2023-12-21

**Authors:** Brianna Barnett, Hailey Minet, Alexander Bishop, Kevin Randall

**Affiliations:** Oklahoma State University, Stillwater, Oklahoma, United States; Oklahoma State University, Stillwater, Oklahoma, United States; Oklahoma State University, Stillwater, Oklahoma, United States; Sam Houston State University, Huntsville, Texas, United States

## Abstract

This study assessed the interconnection between miscarriage, forgiveness, and social provisions on positive valuation of life among N = 157 older women (45 years and older) under correctional custody in Oklahoma. Based on the Model of Developmental Adaptation, path analysis was conducted positing valuation of life, the developmental outcome, regressed on the proximal influence of socio-emotional support and forgiveness and the distal lifetime report of miscarriage. Endogenous measures were controlled for age, race, marital status, education, and crime type. Using Mplus 8.8 and an estimator (MLR) that provides parameter estimates and a chi-square test statistic robust to non-normality, the model fit well with a non-significant chi square test statistics and significant parameter estimates. For these women, 40.5% of the variation in positive valuation of life was explained, primarily by socio-emotional support. Miscarriage had a significant positive association with socio-emotional provisions (β = .15, p < .05) but not forgiveness. Meanwhile, forgiveness had a significant direct association with positive valuation of life (β = .46, p < .001). A significant indirect effect emerged for miscarriage on positive valuation of life through socio-emotional support (.047, p =.035, one-tail test). Results suggest that older incarcerated women with a reported history of miscarriage achieve a positive life outlook to the extent they feel emotionally supported. Greater engagement in forgiveness also appears to contribute to a positive life perspective. This has implications relative to how correctional counselors, social workers, and case managers offer services to support older incarcerated women with a reported history of miscarriage.

